# Structural brain alterations associated with suicidal thoughts and behaviors in young people: results from 21 international studies from the ENIGMA Suicidal Thoughts and Behaviours consortium

**DOI:** 10.1038/s41380-022-01734-0

**Published:** 2022-09-07

**Authors:** Laura S. van Velzen, Maria R. Dauvermann, Lejla Colic, Luca M. Villa, Hannah S. Savage, Yara J. Toenders, Alyssa H. Zhu, Joanna K. Bright, Adrián I. Campos, Lauren E. Salminen, Sonia Ambrogi, Rosa Ayesa-Arriola, Nerisa Banaj, Zeynep Başgöze, Jochen Bauer, Karina Blair, Robert James Blair, Katharina Brosch, Yuqi Cheng, Romain Colle, Colm G. Connolly, Emmanuelle Corruble, Baptiste Couvy-Duchesne, Benedicto Crespo-Facorro, Kathryn R. Cullen, Udo Dannlowski, Christopher G. Davey, Katharina Dohm, Janice M. Fullerton, Ali Saffet Gonul, Ian H. Gotlib, Dominik Grotegerd, Tim Hahn, Ben J. Harrison, Mengxin He, Ian B. Hickie, Tiffany C. Ho, Frank Iorfino, Andreas Jansen, Fabrice Jollant, Tilo Kircher, Bonnie Klimes-Dougan, Melissa Klug, Elisabeth J. Leehr, Elizabeth T. C. Lippard, Katie A. McLaughlin, Susanne Meinert, Adam Bryant Miller, Philip B. Mitchell, Benson Mwangi, Igor Nenadić, Amar Ojha, Bronwyn J. Overs, Julia-Katharina Pfarr, Fabrizio Piras, Kai G. Ringwald, Gloria Roberts, Georg Romer, Marsal Sanches, Margaret A. Sheridan, Jair C. Soares, Gianfranco Spalletta, Frederike Stein, Giana I. Teresi, Diana Tordesillas-Gutiérrez, Aslihan Uyar-Demir, Nic J. A. van der Wee, Steven J. van der Werff, Robert R. J. M. Vermeiren, Alexandra Winter, Mon-Ju Wu, Tony T. Yang, Paul M. Thompson, Miguel E. Rentería, Neda Jahanshad, Hilary P. Blumberg, Anne-Laura van Harmelen, ENIGMA Suicidal Thoughts and Behaviours Consortium, Laura S. van Velzen, Nic J. A. van der Wee, Steven J. van der Werff, Anne-Laura van Harmelen, Lianne Schmaal

**Affiliations:** 1 Orygen, Parkville, VIC Australia; 2 Centre for Youth Mental Health, University of Melbourne, Melbourne, VIC Australia; 3 Department of Psychiatry, University of Cambridge, Cambridge, UK; 4 Institute for Mental Health, School of Psychology, University of Birmingham, Birmingham, UK; 5 McGovern Institute for Brain Research, Massachusetts Institute of Technology, Cambridge, MA USA; 6 Department of Psychiatry, Yale School of Medicine, New Haven, CT USA; 7 Department of Psychiatry and Psychotherapy, Jena University Hospital, Jena, Germany; 8 German Center for Mental Health, Halle-Jena-Magdeburg, Germany; 9 Department of Psychiatry, University of Oxford, Oxford, UK; 10 Melbourne Neuropsychiatry Centre, Department of Psychiatry, The University of Melbourne, Melbourne, VIC Australia; 11 Imaging Genetics Center, Mark and Mary Stevens Neuroimaging and Informatics Institute, Keck School of Medicine, University of Southern California, Marina del Rey, CA USA; 12 Social Genetic & Developmental Psychiatry Centre, Institute of Psychiatry, Psychology & Neuroscience, King’s College London, London, UK; 13 Department of Genetics & Computational Biology, QIMR Berghofer Medical Research Institute, Brisbane, QLD Australia; 14 School of Biomedical Sciences, Faculty of Medicine, University of Queensland, Brisbane, QLD Australia; 15 Institute for Molecular Bioscience, The University of Queensland, St Lucia, QLD Australia; 16 Laboratory of Neuropsychiatry, IRCCS Santa Lucia Foundation, Rome, Italy; 17 Department of Psychiatry, Marqués de Valdecilla University Hospital, IDIVAL, School of Medicine, University of Cantabria, Santander, Spain; 18 Centro Investigación Biomédica en Red de Salud Mental (CIBERSAM), Sevilla, Spain; 19 Department of Psychiatry and Behavioral Sciences, University of Minnesota Medical School, Minneapolis, MN USA; 20 University Clinic for Radiology, University of Münster, Münster, Germany; 21 Center for Neurobehavioral Research, Boys Town National Research Hospital, Boys Town, NE USA; 22 Department of Psychiatry and Psychotherapy, Marburg University, Marburg, Germany; 23 CMBB, Marburg, Germany; 24 Department of Psychiatry, First Affiliated Hospital of Kunming Medical College, Kunming, China; 25 Yunnan Province Clinical Research Center for Psychiatry, Kunming, China; 26 MOODS Team, CESP, INSERM U1018, Faculté de Médecine, Univ Paris-Saclay, Le Kremlin Bicêtre, 94275 France; 27 Service Hospitalo-Universitaire de Psychiatrie de Bicêtre, Hôpitaux Universitaires Paris-Saclay, Assistance Publique-Hôpitaux de Paris, Hôpital de Bicêtre, Le Kremlin Bicêtre, F-94275 France; 28 Department of Biomedical Sciences, Florida State University, Tallahassee, FL USA; 29 Paris Brain Institute (ICM), Inserm (U1127), CNRS (UMR 7225), Sorbonne University, Inria Paris (Aramis project-team), Paris, France; 30 Virgen del Rocío University Hospital, IBiS, CSIC, University of Sevilla, Sevilla, Spain; 31 Institute for Translational Psychiatry, University of Münster, Münster, Germany; 32 Department of Psychiatry, The University of Melbourne, Melbourne, VIC Australia; 33 Neuroscience Research Australia, Randwick, NSW Australia; 34 School of Medical Sciences, University of New South Wales, Kensington, NSW Australia; 35 SoCAT Lab, Department of Psychiatry, School of Medicine, Ege University, Izmir, Turkey; 36 Department of Psychology, Stanford University, Stanford, CA USA; 37 Brain and Mind Centre, University of Sydney, Camperdown, NSW Australia; 38 Department of Psychiatry and Behavioral Sciences, University of California San Francisco, San Francisco, CA USA; 39 Weill Institute for Neurosciences, University of California San Francisco, San Francisco, CA USA; 40 Core-Facility Brainimaging, Faculty of Medicine, University of Marburg, Marburg, Germany; 41 Université de Paris & GHU Paris Psychiatrie et Neurosciences, Paris, France; 42 McGill University, Department of Psychiatry, Montréal, QC Canada; 43 Academic Hospital (CHU), Nîmes, France; 44 University of Minnesota, Department of Psychology, Minneapolis, MN USA; 45 Department of Psychiatry and Behavioral Sciences, Dell Medical School, University of Texas at Austin, Austin, TX USA; 46 Institute of Early Life Adversity Research, Dell Medical School, University of Texas at Austin, Austin, TX USA; 47 Waggoner Center for Alcohol and Addiction Research, University of Texas at Austin, Austin, TX USA; 48 Mulva Clinic for Neuroscience, Dell Medical School, University of Texas at Austin, Austin, TX USA; 49 Department of Psychology, Harvard University, Cambridge, MA USA; 50 Institute for Translational Neuroscience, University of Münster, Münster, Germany; 51 Mental Health Risk and Resilience Research Program, RTI International, Research Triangle Park, NC USA; 52 Department of Psychology and Neuroscience, University of North Carolina at Chapel Hill, Chapel Hill, NC USA; 53 School of Psychiatry, University of New South Wales, Kensington, NSW Australia; 54 Center Of Excellence On Mood Disorders, The University of Texas-Health Science Center at Houston, Houston, TX USA; 55 Louis A. Faillace, MD, Department of Psychiatry and Behavioral Sciences at McGovern Medical School, The University of Texas - Health Science Center at Houston, Houston, TX USA; 56 Center for Neuroscience, University of Pittsburgh, Pittsburgh, PA USA; 57 Center for the Neural Basis of Cognition, University of Pittsburgh, Pittsburgh, PA USA; 58 Department of Child & Adolescent Psychiatry, Psychosomatics and Psychotherapy, University Hospital Münster, Münster, Germany; 59 Department of Psychiatry and Behavioral Sciences, Baylor College of Medicine, Houston, TX USA; 60 Department of Psychology, University of Pittsburgh, Pittsburgh, PA USA; 61 Department of Radiology, IDIVAL, Marqués de Valdecilla University Hospital, Santander, Spain; 62 Advanced Computing and e-Science, Instituto de Física de Cantabria (UC-CSIC), Santander, Spain; 63 Department of Psychiatry, Leiden University Medical Center, Leiden, The Netherlands; 64 Leiden Institute for Brain and Cognition, Leiden University, Leiden, The Netherlands; 65 Leids Universitair Behandel- en Expertise Centrum, Leiden, The Netherlands; 66 Child and Adolescent Psychiatry Leiden University Medical Center, Leiden, The Netherlands; 67 Youz: Child and Adolescent Psychiatry, Leiden, The Netherlands; 68 Department of Psychiatry and Behavioral Sciences, Division of Child and Adolescent Psychiatry, Weill Institute for Neurosciences, UCSF, San Francisco, CA USA; 69 Department of Radiology and Biomedical Imaging, Yale School of Medicine, New Haven, CT USA; 70 Child Study Center, Yale School of Medicine, New Haven, CT USA; 71 Social Security and Resilience Programme, Education and Child Studies, Leiden University, Leiden, The Netherlands

**Keywords:** Depression, Neuroscience, Bipolar disorder

## Abstract

Identifying brain alterations associated with suicidal thoughts and behaviors (STBs) in young people is critical to understanding their development and improving early intervention and prevention. The ENIGMA Suicidal Thoughts and Behaviours (ENIGMA-STB) consortium analyzed neuroimaging data harmonized across sites to examine brain morphology associated with STBs in youth. We performed analyses in three separate stages, in samples ranging from most to least homogeneous in terms of suicide assessment instrument and mental disorder. First, in a sample of 577 young people with mood disorders, in which STBs were assessed with the Columbia Suicide Severity Rating Scale (C-SSRS). Second, in a sample of young people with mood disorders, in which STB were assessed using different instruments, MRI metrics were compared among healthy controls without STBs (HC; *N* = 519), clinical controls with a mood disorder but without STBs (CC; *N* = 246) and young people with current suicidal ideation (*N* = 223). In separate analyses, MRI metrics were compared among HCs (*N* = 253), CCs (*N* = 217), and suicide attempters (*N* = 64). Third, in a larger transdiagnostic sample with various assessment instruments (HC = 606; CC = 419; Ideation = 289; HC = 253; CC = 432; Attempt=91). In the homogeneous C-SSRS sample, surface area of the frontal pole was lower in young people with mood disorders and a history of actual suicide attempts (*N* = 163) than those without a lifetime suicide attempt (*N* = 323; FDR-*p* = 0.035, Cohen’s *d* = 0.34). No associations with suicidal ideation were found. When examining more heterogeneous samples, we did not observe significant associations. Lower frontal pole surface area may represent a vulnerability for a (non-interrupted and non-aborted) suicide attempt; however, more research is needed to understand the nature of its relationship to suicide risk.

## Introduction

Suicide is the second leading cause of death for young people aged between 15 and 29 [1]. Suicidal thoughts and behaviors (STBs) typically emerge during adolescence [2]. It has been estimated that between 11 and 29% of adolescents report suicidal ideation (suicidal thoughts), and 2–10% of adolescents attempted suicide in the past year [3]. Unfortunately, the number of suicide attempts among children and adolescents has continued to increase sharply despite national and international prevention efforts [4].

To improve targeting of prevention and intervention efforts and thereby reduce the number of deaths by suicide in this age group, we must increase our understanding of the mechanisms underlying both suicidal thoughts and suicidal behaviors (including suicide attempts) in young people. Neuroimaging, including Magnetic Resonance Imaging (MRI), is a useful tool with which to identify biological risk markers for STBs in vivo and non-invasively. Many neuroimaging studies have been published examining the neural substrates of STBs in the past 20 years, but few have focused on STBs in youth (for a review, see [5]). Although several of these studies support lower regional brain volumes, particularly in ventral and dorsal prefrontal and also in temporal regions [6–9] in suicide attempters with mood disorders, negative findings have also been reported [10, 11]. Structural brain alterations related to suicidal ideation in young people have inconsistently been reported in the striatum and temporal lobes [12–14].

In addition to the small number of studies focusing on youth, neuroimaging studies investigating associations between structural brain measures and STBs have also been limited by small sample sizes [5]. There are multiple limitations associated with small sample sizes. First, small sample sizes decrease power, increase the probability of false-negative effects, and inflate the effect size estimate when an actual effect is observed [15]. Second, there may be small yet clinically significant associations between STBs and brain structure. To reliably identify these effects, larger samples are needed. Another significant limitation of previous work is that clinical controls (CC) are often not included, making it difficult to understand if alterations are specific to STBs or reflect mental health disorders in general [5].

To address these limitations, the suicide project within the ENIGMA Major Depressive Disorder (ENIGMA-MDD) consortium pooled data from 18 different studies worldwide to examine associations between brain morphology and suicide attempt in major depressive disorder (MDD) patients [16, 17]. Findings showed a lower volume of the thalamus and pallidum and a smaller surface area of the inferior parietal lobe in adults with MDD and a history of suicide attempts (*N* = 679) compared to individuals with MDD without a history of suicide attempt (*N* = 5484). However, these studies did not examine structural MRI correlates of suicidal ideation. In addition, studies within ENIGMA-MDD are limited to individuals with MDD, while STBs are transdiagnostic phenomena, and the extent of neurobiological mechanisms underlying STBs that are common to or may differ across psychiatric disorders is unknown. Finally, these previous studies did not examine structural brain alterations in children and adolescents.

Therefore, we established the transdiagnostic ENIGMA Suicidal Thoughts and Behaviours (ENIGMA-STB) consortium, which allows investigation of neural correlates of STBs across a range of psychiatric conditions, leveraging many samples worldwide. This large dataset enables assessment of structural brain alterations that are common across groups (e.g., groups with a variety of psychiatric conditions including mood disorders, anxiety disorders, post-traumatic stress disorder, addiction, and obsessive-compulsive disorder), and also alterations that are specific to subgroups, such as males or females. For this ENIGMA-STB study, we focused specifically on young persons, as there is limited information concerning the mechanisms underlying STB in this group.

**Fig. 1:**
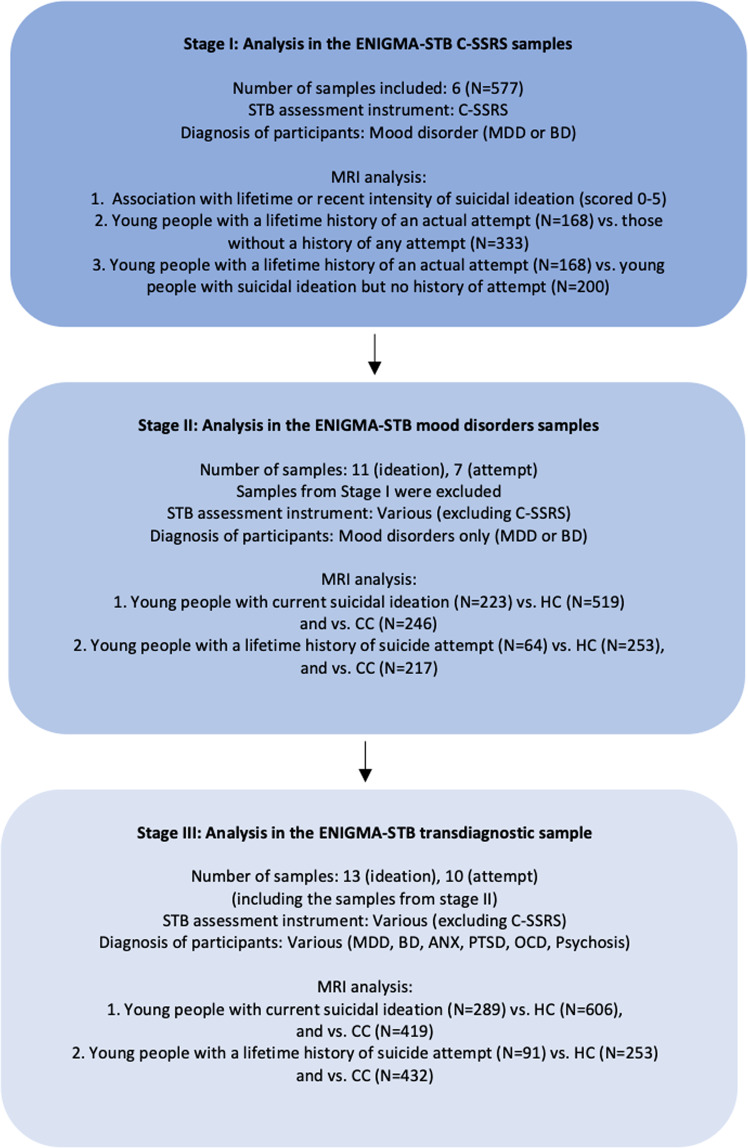
Overview of the three stages of the analysis. The color indicates the homogeneity of the samples (dark blue=most homogeneous in terms of STB assessment instruments and type of psychiatric disorders, light blue=most heterogeneous in terms of STB assessment instruments and type of psychiatric disorders). C-SSRS Columbia Suicide Severity Rating Scale; HC healthy controls; CC clinical controls.

As we expected the effect sizes would be small due to clinical heterogeneity and use of different instruments to assess STBs, we started with the most homogeneous sample in terms of assessment instruments and type of psychiatric disorder (i.e., mood disorders). These six homogeneous samples were enriched for STBs and conducted a more in-depth assessment of STBs (e.g., not only the presence but also the intensity of suicidal ideation) by use of the Columbia Suicide Severity Rating Scale. Here we aimed to investigate differences in structural MRI measures between young persons with a lifetime history of suicide attempt compared to those without and examine associations with the intensity of suicidal ideation. We next evaluated associations between MRI metrics and suicidal thoughts and behavior in a larger sample with mood disorders but more heterogeneity in assessment instruments. In this sample we aimed to identify structural brain alterations in young persons with (1) a lifetime history of a suicide attempt; and (2) current (in the past week, 2 weeks, or month) suicidal ideation (but no history of attempt), compared to healthy controls (HC) and CC. Finally, we examined these associations in the largest sample including youth ENIGMA STB samples with heterogeneity in both diagnosis and assessment instruments (see Fig. 1). Based on previous findings in adolescents, we predicted that STBs would be associated with structural alterations in the prefrontal cortex (PFC) [6, 8, 9], temporal cortex [12], and caudate [14].

## Patients and methods

### Samples

This mega-analysis included data from 21 international studies from ten countries to examine the association between STBs and brain structure in young people ages 8–25 years. The inclusion/exclusion criteria for the different studies are presented in Table S1. All sites obtained ethics approval from their local institutional review boards and ethics committees. All participants who were 18 years old and over provided written informed consent, and those aged under age 18 years provided written informed assent in addition to written informed consent from a parent/guardian at the local institution.

### Image processing and harmonization

Structural T1-weighted brain MRI scans were acquired at each site. Information regarding the acquisition parameters, software versions, and scanner characteristics for the different sites is presented in Table S2. The T1-weighted images were analyzed locally using harmonized analysis and quality control protocols for FreeSurfer [18] (http://surfer.nmr.mgh.harvard.edu/), developed by the ENIGMA consortium (http://enigma.ini.usc.edu/protocols/imaging-protocols/). The ENIGMA FreeSurfer protocol provides tools for quality control of the segmented cortical and subcortical phenotypes. Each site visually inspected the segmentation and excluded regions that were not appropriately segmented. To reduce the number of statistical tests and avoid issues related to left-right flipping that may have occurred at the various sites, we combined regional measures across both hemispheres by taking the mean of the left and right hemisphere regions. We examined the volume of eight subcortical regions and cortical thickness and surface area of 34 regions, defined by the Desikan-Killiany atlas [19]. In addition, two global measures were calculated: mean cortical thickness and total surface area across both hemispheres, creating a total of 78 brain measures.

Before the statistical analysis, neuroimaging measures were harmonized across sites using the ComBat algorithm in R [20, 21], with age, sex, and psychiatric diagnosis as covariates. ComBat uses an empirical Bayes approach to adjust for variability between scanners while still preserving biological variability related to age, sex, and diagnosis. All brain measures included in the statistical analyses were ComBat-corrected. After correction, within-site outliers (measures greater than three standard deviations away from the mean of that region) were excluded from the analysis.

### Statistical analysis

As we anticipated small effect sizes due to heterogeneity in diagnosis and instruments used to assess STBs, we performed the analyses in three separate stages, moving from homogeneous samples to more heterogeneous samples (please see Fig. 1 and the description of analyses per stage below). All reported p-values were corrected for multiple comparisons (for the 78 brain measures) using the Benjamini Hochberg correction in R to ensure an FDR < 0.05. 95% confidence intervals are reported for all analyses in supplemental tables.

### Stage I: Analysis in the ENIGMA-STB Columbia Suicide Severity Rating Scale (C-SSRS) sample

We first examined associations between brain structure and STBs in a subsample of six cohorts, all of which used an instrument designed specifically to assess suicidal ideation and suicide attempt, the C-SSRS (see Table 1). The C-SSRS is a reliable and well-validated interview, specifically developed to assess intensity and severity of suicidal thoughts, and suicidal behavior [22]. These cohorts included participants with MDD or bipolar disorder (BD) diagnoses (*N* = 577, age range 11–25) (HC samples were excluded from analyses due to no or limited C-SSRS data). Multiple linear regression analyses were conducted in R, and age, sex, and age-by-sex interactions were included as covariates in all analyses. Intracranial volume (ICV) was included as an additional covariate in analyses of subcortical volume and cortical surface area. Because we had estimated and controlled for the contribution of site and scanner using ComBat prior to conducting the analysis (see above), these measures were not included as covariates. In the regression models, the structural brain measures were included as dependent variables. For suicidal ideation analyses, the continuous C-SSRS measure of recent and lifetime intensity of suicidal ideation were included as predictors. This variable was coded 0–5 (0: no ideation; 1: passive ideation; 2: non-specific active ideation; 3: active ideation with a method, but no plan or intent; 4: active ideation with intent, but no plan; 5: active ideation with a plan and intent). We then examined differences in brain morphology between young people with a lifetime history of any attempt (actual, aborted or interrupted attempts) and young people with no lifetime history of attempt.

**Table 1:** Descriptive statistics for studies included in the C-SSRS sample.

Site	Main diagnosis in sample	Age (years)	% Female	Total *N*
Melbourne (YODA)	MDD	19.0 range 15–25	57.6	139
MR-IMPACT	MDD	15.0 range 11–17	76.1	113
Stanford TAD	MDD	16.5 range 14–18	76.2	42
Stanford TIGER	MDD	16.0 range 13–18	67.6	34
UCSF	MDD	16.0 range 13–18	63.4	71
Yale School of Medicine	MDD + BD	19.0 range: 13–25	64.0	178
Total	MDD + BD	17.0 range 11–25	65.9	577

Presented here are age (median, minimum-maximum) and sex for the six sites in the C-SSRS sample.

*MDD* major depressive disorder, *BD* bipolar disorder.

We also examined differences in brain structure between individuals with a lifetime history of an actual suicide attempt (but not interrupted or aborted attempts) and those without any lifetime attempt. We examined actual attempts and did not include interrupted or aborted attempts, as previous work suggests that actual suicide attempt may represent a more clinically severe and reliable phenotype than interrupted and aborted attempts [23, 24]. Finally, we compared brain morphology between individuals with a lifetime history of suicidal ideation (but no lifetime history of an actual attempt) and those with a lifetime history of an actual attempt. In secondary analyses, we examined the difference in brain structure between individuals with a lifetime history of any attempt (aborted, interrupted or actual attempt) compared to those without any attempt (see Supplemental Note 3 for a description and findings). Effect size estimates were calculated using the Cohen’s *d* metric for group comparisons and the standardized beta for associations with the continuous recent or lifetime intensity of suicidal ideation measure.

#### Stage II: Analysis in the ENIGMA-STB mood disorders samples

We subsequently examined associations between STBs and brain structure in a combined sample of cohorts that assessed STBs using various instruments other than the C-SSRS. For demographic characteristics of these cohorts, please see Tables 2A and 3A. This larger sample (which did not include the six cohorts from Stage I) included HC and individuals with a current or lifetime diagnosis of MDD or BD. Various instruments were used to assess current suicidal ideation and lifetime history of suicide attempts across cohorts. An overview of these instruments is presented in Table S3, and the approach used to harmonize these measures across cohorts is described in Supplemental Note 1. In short, history of lifetime suicidal attempt (yes/no) was determined using diagnostic interviews [e.g., 25, 26]. Current suicidal ideation (in the past week, 2 weeks or month; yes/no) was determined using a diagnostic interview, or items from depression severity rating scales [e.g., [27, 28]. Because only five sites had information on both suicidal ideation and suicide attempt, and previous work in adults has documented differences between the neural correlates of ideation and attempt [29], we conducted separate analyses for suicidal ideation and suicide attempt to optimize the sample size for each analysis. To examine suicidal attempts, we compared three groups: 1) HC, without a current or lifetime psychiatric diagnosis or lifetime history of suicide attempt (“healthy controls”); 2) “clinical controls”, with a current or lifetime mood disorder, but no lifetime history of suicide attempt and 3) “clinical attempters”; young people with a current or lifetime mood disorder and lifetime history of suicide attempt. To examine current suicidal ideation, we created three groups: 1) HC without a current or lifetime psychiatric diagnosis or lifetime history of suicide attempt or current suicidal ideation; 2) CC with a current or lifetime mood disorder but no current suicidal ideation or lifetime history of suicide attempt; and 3) young people with a current or lifetime mood disorder and current suicidal ideation, but no lifetime history of suicide attempt.

**Table 2:** Descriptive statistics for studies included in the ideation analysis.

Site	Age HC (years)	Age CC (years)	Age ideation	% female HC	% female CC	% female ideation	Total *N* HC	Total *N* CC	Total *N* Ideation
*A. Mood disorders only sample*									
Boystown (USA)	17.0 (14–18)	17.0 (13–19)	17.0 (14–18)	22.2	60.0	54.4	9	10	11
DEP-ARREST-CLIN – MOODS (France)	21.0 (20–25)	-	22.0 (18–25)	54.5	-	70.0	11	0	10
EPISCA (Netherlands)	14.0 (13–19)	15.5 (13–16)	16.0 (13–20)	86.2	83.3	88.9	29	6	18
FOR2107-Marburg	23.0 (18–25)	24.0 (18–25)	23.0 (18–25)	67.2	84.4	49.1	125	32	57
FOR2107-Münster (Germany)	23.0 (18–25)	22.0 (18–25)	23.0 (19–25)	71.7	77.3	50.0	106	22	26
Houston BD (USA)	14.0 (8–25)	14.0 (8–25)	14.0 (10–24)	54.3	31.6	66.7	81	57	9
MDD Cohort (China)	23.0 (22–24)	22.0 (18–25)	21.0 (16–25)	80.0	44.4	66.7	5	9	12
Muenster Neuroimaging Cohort (Germany)	22.0 (17–25)	23.0 (16–25)	22.0 (17–25)	47.5	57.9	50.0	80	19	40
Sydney Brain and Mind Centre (Australia)	23.0 (18–25)	19.5 (12–25)	17.0 (15–22)	60.0	61.4	77.8	25	44	9
University of Minnesota (USA)	16.0 (12–19)	16.0 (13–20)	16.0 (12–19)	54.5	74.3	84.0	22	35	25
University of Texas- Austin -Bipolar Seed Program (USA)	21.0 (18–25)	21.0 (18–25)	20.5 (19–25)	69.2	75.0	83.3	26	12	6
Total	22.0 (8–25)	19.0 (8–25)	20.0 (10–25)	62.4	61.0	61.4	519	246	223
*B. Transdiagnostic sample*									
Boystown (USA)	17.0 (14–18)	16.0 (12–19)	16.0 (12–18)	22.2	30.7	63.0	9	114	27
DEP-ARREST-CLIN – MOODS (France)	21.0 (20–25)		22.0 (18–25)	54.5		70.0	11	0	10
EPISCA (Netherlands)	14.0 (13–19)	16.0 (13–20)	16.0 (12–20)	86.2	83.3	87.5	29	12	24
FOR2107-Marburg (Germany)	23.0 (18–25)	24.0 (18–25)	23.0 (18–25)	67.2	84.4	49.1	125	32	57
FOR2107-Münster (Germany)	23.0 (18–25)	22.0 (18–25)	23.0 (19–25)	71.7	77.3	50.0	106	22	26
Houston BD (USA)	14.0 (8–25)	14.0 (8–25)	14.0 (10–24)	54.3	31.6	66.7	81	57	9
MDD Cohort (China)	23.0 (22–24)	22.0 (18–25)	21.0 (16–25)	80.0	44.4	66.7	5	9	12
Muenster Neuroimaging Cohort (Germany)	22.0 (17–25)	23.0 (16–25)	22.0 (17–25)	47.5	57.9	50.0	80	19	40
SOCAT (Turkey)	23.0 (17–25)		23.0 (19–25)	100.0		95.0	37	0	20
Sydney Brain and Mind Centre (Australia)	23.0 (18–25)	19.0 (12–25)	20.0 (14–25)	60.0	61.4	85.7	25	83	21
University of Minnesota (USA)	16.0 (12–19)	16.0 (13–20)	16.0 (12–19)	54.5	74.3	84.0	22	35	25
University of Texas- Austin -Bipolar Seed Program (USA)	21.0 (18–25)	21.0 (18–25)	20.5 (19–25)	69.2	75.0	83.3	26	12	6
UWashington/Harvard (USA)	11.0 (8–16)	10.5 (8–16)	14.5 (8–16)	50.0	50.0	58.3	50	24	12
Total	21.0 (8–25)	17.0 (8–25)	20.0 (8–25)	63.7	52.5	65.7	606	419	289

Presented here are age (median, minimum, maximum) and sex for the three groups (HC healthy controls, CC clinical controls, Ideation: group with current suicidal ideation) for the different sites included in the analysis on suicidal ideation in the stage II mood disorder only sample (A) and stage III transdiagnostic sample (B).

**Table 3:** Descriptive statistics for sites included in the attempt analysis.

Site	Age HC (years)	Age CC (years)	Age Attempt	% female HC	% female CC	% female Attempt	Total *N* HC	Total *N* CC	Total *N* Attempt
*A. Mood disorders only sample*									
DEP-ARREST-CLIN - MOODS (France)	-	22.0 (18–25)	19.0 (18–23)	-	70.0	55.6	0	10	9
Houston BD (USA)	14.0 (8–25)	15.0 (8–25)	17.0 (11–24)	48.5	34.7	69.2	97	75	13
Sydney Bipolar Kids and Siblings	21.0 (13–25)	22.5 (16–25)	22.0 (18–25)	50.0	62.5	66.7	64	16	9
Sydney Brain and Mind Centre (Australia)	-	19.5 (15–25)	20.5 (15–24)	-	66.7	100.0	0	48	8
University of Minnesota (USA)	16.0 (14–20)	16.0 (12–19)	17.5 (12–19)	66.7	78.0	62.5	12	41	8
University of Texas- Austin - Bipolar Seed Program (USA)	21.0 (18–25)	21.0 (18–25)	21.0 (19–25)	69.2	77.8	66.7	26	18	9
UWashington/Harvard (USA)	11.5 (8–16)	15.0 (12–16)	14.5 (9–17)	53.7	55.6	75.0	54	9	8
Total	16.0 (8–25)	17.0 (8–25)	19.0 (9–25)	53.0	58.1	70.3	253	217	64
*B. Transdiagnostic sample*									
DEP-ARREST-CLIN - MOODS (France)		22.0 (18–25)	19.0 (18–23)		70.0	55.6	0	10	9
Fondazione Santa Lucia - Schizophrenia sample (Italy)		23.0 (16–25)	24.0 (20–25)		5.3	57.1	0	19	7
Houston BD (USA)	14.0 (8–25)	15.0 (8–25)	17.0 (11–24)	48.5	34.7	69.2	97	75	13
PAFIP1 (Spain)		22.0 (17–25)	22.0 (19–24)		25.5	14.3	0	51	7
PAFIP2 (Spain)		21.0 (17–25)	22.5 (17–25)		24.1	50.0	0	58	10
Sydney Bipolar Kids and Siblings (Australia)	21.0 (13–25)	22.0 (16–25)	22.0 (18–25)	50.0	60.9	66.7	64	23	9
Sydney Brain and Mind Centre (Australia)		19.0 (13–25)	19.0 (15–24)		68.3	100	0	101	10
University of Minnesota (USA)	16.0 (14–20)	16.0 (12–19)	17.5 (12–19)	66.7	78.0	62.5	12	41	8
University of Texas- Austin - Bipolar Seed Program (USA)	21.0 (18–25)	21.0 (18–25)	21.0 (19–25)	69.2	77.8	66.7	26	18	9
UWashington/Harvard (USA)	11.5 (8–16)	12.5 (8–16)	13.0 (9–17)	53.7	52.8	77.8	54	36	9
Total	16.0 (8–25)	19.5 (8–25)	20.0 (9–25)	53.0	48.4	63.7	253	432	91

Presented here are age (median, minimum-maximum) and sex for the three groups (HC healthy controls, CC clinical controls, attempt: group with lifetime suicide attempt) for the different sites included in the analysis on lifetime history of suicide attempts in the stage II mood disorder only sample (A) and stage III transdiagnostic sample (B).

Similar to the analyses in the C-SSRS sample, group differences in subcortical volume, cortical thickness, and cortical surface area were compared using multiple linear regression models in R. Because we were specifically interested in differences between individuals with current suicidal ideation or past suicide attempt(s) versus HC or CC, we included a group predictor variable to compare the suicide attempt group to either CC or to HC (in two-group comparisons). In analyses of current suicidal ideation, a group predictor was included to compare the ideation group to either CC or HC. Covariates in the models included age, sex, and age-by-sex interaction. In addition, we corrected for ICV when analyzing subcortical volumes and cortical surface area measures. We calculated effect size estimates using Cohen’s *d* metric.

#### Stage III: Analysis in the ENIGMA-STB transdiagnostic sample

To further investigate the effect of heterogeneity related to type of diagnosis and to provide additional power to potentially detect any differences not identified in the analyses restricted to mood disorders in stage II, we examined the correlates of current suicidal ideation and lifetime history of suicide attempt in a transdiagnostic sample from multiple international cohorts (*N* cohorts for the ideation analysis = 13; *N* cohorts for the attempt analysis = 10). For demographic characteristics of these cohorts, please see Tables 2B and 3B. This transdiagnostic sample included the cohorts included in stage II, with additional cohorts of individuals with mental disorders other than MDD or BD. The analyses performed were similar to the analyses performed in stage II. Given the large sample size of this sample, we were able to conduct additional analyses, and examine subgroups. We conducted the above-mentioned analyses in this larger transdiagnostic ENIGMA-STB sample separately for males and females, including age and ICV as covariates. Data on lifetime psychiatric diagnosis were available in a subsample of participants (*N* = 371 in the ideation analysis and *N* = 380 in the attempt analysis), as some sites only assessed current disorders. Therefore, in secondary analyses, diagnosis type was included as an additional covariate (see Supplemental Note 2).

## Results

### Stage I: Associations with suicidal ideation and attempts in the ENIGMA-STB C-SSRS sample

**Fig. 2:**
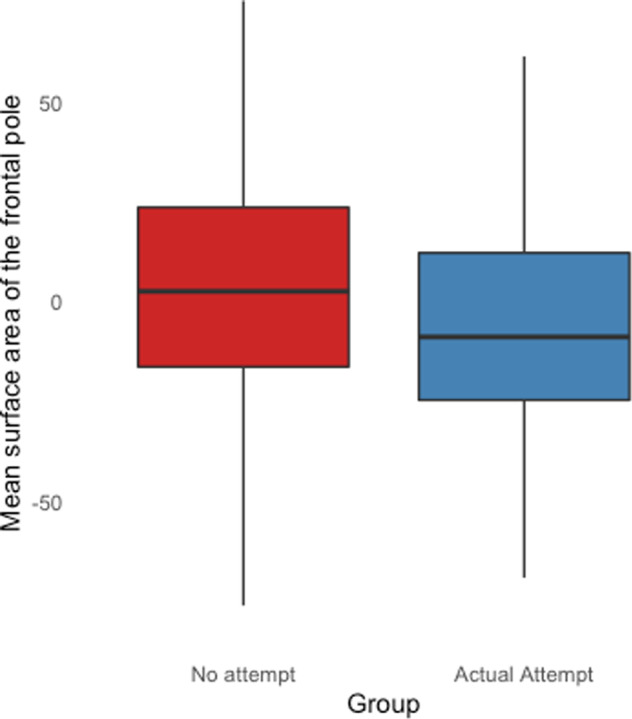
Boxplot showing the mean surface area of the frontal pole in young people without a lifetime history of any suicide attempt (in red), and those with a lifetime history of an actual suicide attempt (in blue). Lifetime history of an actual suicide attempt was assessed using the C-SSRS.

There were no significant associations between lifetime or recent intensity of ideation and cortical thickness, cortical surface area, and subcortical volume measures (*N* = 438 and 510 respectively; Tables S4 and S5). Surface area of the frontal pole was lower in young people with a lifetime history of an actual suicide attempt (*N* = 163) compared to those with no lifetime attempt history (*N* = 323; FDR *p* value = 0.035; Cohen’s *d*:−0.342; lower bound CI: −0.531; upper bound CI: −0.152; Table S6 and Fig. 2). Finally, there were no significant differences between those with lifetime ideation (but no history of a prior actual suicide attempt) (*N* = 200) and those with a lifetime history of an actual attempt (*N* = 168; Table S7).

### Stage II: Associations with current suicidal ideation and history of attempt in the ENIGMA-STB mood disorders samples

#### Current suicidal ideation

In the ENIGMA-STB sample of participants with mood disorders (eleven cohorts; excluding the six C-SSRS samples) in which STBs were assessed using various instruments, no brain structure measure differed significantly between young people with current suicidal ideation (*N* = 223) and HC (*N* = 519; Table S8) or CC (*N* = 246; Table S9) groups.

#### Lifetime history of suicide attempt

In this sample from seven cohorts (the six C-SSRS samples were excluded), MRI measures also did not differ significantly between the suicide attempt group (*N* = 64) and HC (*N* = 253; Table S10) or CC (*N* = 217; Table S11) groups.

### Stage III: Associations with current suicidal ideation and history of attempt in the ENIGMA-STB transdiagnostic sample

#### Current suicidal ideation

In the transdiagnostic ENIGMA-STB sample (not restricted to MDD or BD diagnosis; 13 cohorts) no brain structure measure differed significantly between young people with current suicidal ideation (*N* = 289) and HC (*N* = 606; Table S12) or CC (*N* = 419; Table S13) groups. No differences were observed when additionally adjusting for primary diagnosis type (Table S14), nor when conducting separate analyses in males and females (*N* HC = 145, *N* CC = 109, *N *ideation = 77 in males; *N* HC = 343, *N* CC = 181, *N* ideation = 146 in females; Tables S15, S16, S17 and S18).

#### Lifetime history of suicide attempt

In the larger transdiagnostic ENIGMA-STB sample (not restricted to MDD or BD diagnosis; ten cohorts), MRI measures also did not differ significantly between the suicide attempt group (*N* = 91) and HC (*N* = 253; Table S19) or CC (*N* = 432; Table S20) groups. No differences were observed when correcting for primary diagnosis type (Table S21) or conducting separate sex-stratified analyses (*N* CC = 82, *N* attempt=11 in males; *N* HC = 134, *N* CC = 195, *N* attempt = 53 in females; Tables S22, S23 and S24).

## Discussion

In this study we examined the associations between STBs and structural MRI measures in young people in samples from the ENIGMA-STB consortium. In a homogeneous combined sample (six sites, *N* = 577) assessed with the same well-validated and widely-established instrument specifically developed to assess STBs (C-SSRS) and including only young people with MDD or BD (age range 11–25 years), we found a significantly smaller surface area in the frontal pole in those with a lifetime history of an actual suicide attempt compared to those without any history of attempt.

The frontal pole is the rostral-most aspect of the prefrontal cortex and plays an essential role in higher-order functions involved in emotion and other behavioral regulation, notably, decision-making and cognitive inhibition, as well as social cognition processes (e.g., self-referential processes) implicated in STBs [30–33]. As cortical surface area is highly heritable [34] and is less affected by environmental factors during development and in later life, than is the cortical thickness [35], alterations in frontal pole surface area may represent a pre-existing vulnerability for suicidal behavior in adolescents. Longitudinal studies are needed to elucidate whether structural alterations, in particular cortical surface area, in this region precede the onset of STBs in youth. In a previous longitudinal study, structural alterations in the frontal pole (amongst other frontal regions) were associated with a family history of BD, which is also associated with increased risk of developing STBs [36]. In another longitudinal study of a sample of 46 young people with mood disorders, decreases in rostral prefrontal volume were found to be associated with future suicide attempts, although thickness and surface area were not studied separately [9]). Together with the findings of this study, results suggest that decreases in rostral PFC surface area warrant further study as potential predictors of and targets for the prevention of suicide.

In more heterogeneous samples in terms of diagnosis type or instruments to assess STBs, we did not observe any significant group differences related to ideation or attempt, which may be (partially) due to clinical heterogeneity, and the less specific and consistent definition used for suicide attempts in these samples. In contrast to the C-SSRS, the measures used to assess STBs in these more heterogeneous combined samples, do not distinguish between interrupted or aborted suicide attempts, and actual suicide attempts, therefore the attempt group may have included less severe phenotypes, decreasing our ability to discriminate those who do and do not attempt suicide based on the three MRI metrics examined here. This is supported by a supplementary analysis in the C-SSRS sample (stage I) comparing a group with interrupted, aborted or actual attempt (i.e., not restricted to actual attempt) to those without a history of attempt, which showed a reduction in effect size for the association with surface area of the frontal pole, compared to the analysis including only actual attempters (Cohen’s *d* = −0.295 versus −0.342 respectively; see Supplemental Note 3).

In line with our hypothesis that interrupted or aborted attempts may represent a less severe phenotype, Rogers and colleagues [24] reported less severe clinical symptoms in individuals with a history of an aborted attempt, compared to individuals with an actual attempt. However, a second study [37] did not find differences in symptom severity between individuals with an interrupted or aborted suicide attempt compared to individuals with an actual suicide attempt. A potential alternative explanation for our findings may be that interrupted or aborted attempts are qualitatively different and therefore may have revealed different associations with brain morphology. While there is limited research on this topic, previous work suggests that aborted or interrupted attempts and actual attempts do not differ in terms of lethality or intentionality [38, 39]. Another factor that may play a role in explaining our findings are differences in the reliability of assessing interrupted, aborted or actual suicide attempts. Mundt et al. [23] showed that inter-rater reliability for interrupted or aborted attempt (kappa = 0.48 and 0.89, respectively), may be lower than for actual attempt (kappa = 1.0). Thus variability across ENIGMA-STB cohorts in how interrupted or aborted attempts were coded, may have increased noise and reduced our ability to identify associations between brain structure and suicide attempt, when interrupted or aborted attempts are included in the definition of attempt.

While we observed lower frontal pole surface area to be associated with actual suicide attempts, a recent study that examined the association between STBs and brain structure in over 6000 younger children aged 9–10 years in the Adolescent Brain Cognitive Development (ABCD) study did not reveal significant structural alterations in association with STBs [40]. This may be related to the fact that the ABCD study is a general population sample with only a few children diagnosed with mood disorders, and STBs were less common and severe. Given the important role of puberty-related developmental processes in STBs, the ABCD sample may have been too young to detect brain alterations [41]. In addition, the study did not distinguish between actual attempts, and interrupted or aborted attempts, which may have also decreased the ability to identify significant alterations associated with suicide attempt. A prior ENIGMA-MDD study did find significant differences in brain structure in adults with MDD and a history of suicide attempts, including in the thalamus, pallidum and inferior parietal lobe [17]. However, the previous ENIGMA-MDD suicide study focused on adults and included only people with MDD and HC, whereas here, we included a transdiagnostic sample of young people. We hypothesize that the structural alterations associated with STBs may be stronger in adults, than in children, given the potential for prolonged exposure to stress and reduced neural plasticity in adults [42]. In future work in the ENIGMA-STB consortium, we will be able to test this new hypothesis.

This study shows the importance of using well-validated and detailed phenotyping of STBs, such as the C-SSRS, when pooling data. Therefore, a strength of this study includes the large sample sizes that allowed the examination of more detailed and homogeneous phenotypes. An additional strength of the study is the use of harmonized protocols for image processing and quality control. We should also note a few limitations of this study. First, different instruments were used to assess STBs across cohorts for analyses in the larger ENIGMA-STB samples (stage II and III), although we used a detailed process to harmonize measures across studies. Moreover, when multiple instruments were used to assess suicidal ideation or attempts within one cohort, we defined STBs in that sample using instruments that showed strong correlations with the instruments used by other cohorts to assess STBs [43]. Future multi-site collaborations would be improved by prospective harmonization in data collection and/or measurement. A second limitation was the cross-sectional study design. Although the findings are consistent with a prior report on future suicide attempts [9], we cannot determine whether brain structure increases the risk for STBs or whether prior attempts affect brain structure. Finally, while including participants from many international studies, the samples mainly included Caucasian participants from high-income countries.

In conclusion, by harmonizing neuroimaging data from research groups worldwide, we found that a deficit in the surface area of the frontal pole was related to actual (non-interrupted and non-aborted) suicide attempts in young people with mood disorders, which we interpret may represent a preexisting vulnerability to suicide attempts. Future studies which aim to pool data across studies, require well-validated measures and detailed phenotyping of STBs. Future studies focusing on the frontal pole may elucidate the structural and functional neurobiological mechanisms through which this region contributes to the development of STBs in young people.

## Supplementary information

Supplemental material
